# Pharmacological Effects and Mechanisms of Action of Myricanol

**DOI:** 10.3390/molecules31111781

**Published:** 2026-05-22

**Authors:** Kai He, Hu Li, Han Sun, Ning Li, Tong Wang, Jian-Dong Jiang, Zong-Gen Peng

**Affiliations:** 1 Biomedical Research Institute, Hunan University of Medicine, Huaihua 418000, China; hekai69@126.com; 2 CAMS Key Laboratory of Antiviral Drug Research, Institute of Medicinal Biotechnology, Chinese Academy of Medical Sciences & Peking Union Medical College, Beijing 100050, China; lihu0112@imb.pumc.edu.cn (H.L.);; 3 State Key Laboratory of Bioactive Substance and Function of Natural Medicines, Institute of Medicinal Biotechnology, Chinese Academy of Medical Sciences & Peking Union Medical College, Beijing 100050, China

**Keywords:** myricanol, diarylheptanoid, antioxidant, anticancer, metabolic diseases, anti-inflammation

## Abstract

The bark of *Myrica rubra* (Lour.) Siebold & Zucc (*M. rubra*) is a natural remedy widely used in China and other Asian countries to treat tissue and bone injuries, burns, scalds, gastrointestinal ulcers, and diarrhea. Myricanol is an important ingredient in the bark of *M. rubra*. This review summarizes articles published over the past 26 years on the pharmacological effects and mechanisms of action of myricanol, aiming to advance research and applications of myricanol. Evidence shows that myricanol has multiple bioactive properties, including antioxidant, anticancer, anti-inflammatory, antimicrobial, antidiabetic, and antihyperlipidemic effects. Myricanol improves metabolic abnormalities in mice by activating the AMPK/SIRT1/PGC-1α signaling pathway. It also demonstrates significant anticancer, antioxidant, and anti-inflammatory actions, primarily by regulating Caspase and BCL-2 family proteins, inhibiting iNOS expression, scavenging free radicals, and interacting with Peroxiredoxin 5. Therefore, myricanol shows great potential for the treatment of cancer, metabolic abnormalities, and inflammatory bowel disease. Further research is needed to improve its bioavailability, confirm its pharmacological effects and mechanisms in vivo, and explore its pharmacokinetic properties and safety.

## 1. Introduction

**Figure 1:**
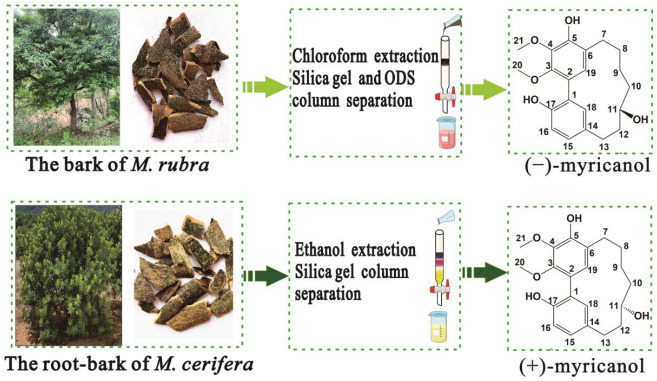
Structure of (±) myricanol isolated from the bark of *M. rubra* and *Myrica cerifera* L. (*M. cerifera*).

Diarylheptanoids are a class of secondary plant metabolites with diverse biological activities. Based on their structural features, diarylheptanoids are mainly classified as linear or cyclic. For example, curcumin is a representative linear diarylheptanoid, well known for its medicinal applications in treating cancer, inflammatory diseases, and skin disorders [1]. As a natural cyclic diarylheptanoid, myricanol is attracting increasing attention as a potential drug candidate for various diseases. Myricanol has a seven-carbon skeleton linked to two phenyl rings; one ring contains two methoxyl groups and one phenolic hydroxyl group, whereas the other ring has only one phenolic hydroxyl group (Figure 1). Structure–activity relationship studies of cyclic diarylheptanoids have shown that the methoxyl and phenolic hydroxyl groups on the phenyl rings may be crucial for the antioxidant and anticancer activities of myricanol [2]. Recently, myricanol has been widely investigated due to its unique structural features and its anti-inflammatory, antioxidant, anticancer, and lipid-lowering activities [3].

As early as 1971, Begley et al. isolated myricanol and myricanone from the stem bark of *Myrica nagi* Thunb (*M*. *nagi*) [4]. Since then, myricanol has been found in other plants, such as the roots of *Pourthiaea villosa* Decne (*P*. *villosa*) [5], *Myrica adenophora* Hance (*M*. *adenophora*), and the stem bark of *Myrica esculenta* Buch. Ham. ex D. Don (*M*. *esculenta*), *Alnus japonica* Thunb. Steud (*A*. *japonica*), *M*. *cerifera*, *M*. *rubra* [6,7], and the root and stem barks of *Myrica arborea* Hutch (*M. arborea*) [8], etc. High-performance liquid chromatography (HPLC) analysis showed that the amounts of myricanol in the stem bark of *M. esculenta* and *M*. *rubra* are 4.78% and 2.18%, respectively [9]. Ting et al. obtained 154 mg of myricanol from 7.2 kg of dried roots of *M. esculenta* [6]. In our previous study, myricanol was isolated by both column chromatography and high-speed countercurrent chromatography from the stem bark of *M*. *rubra* with a 0.04% yield [10].

Currently, the stem bark of Myricaceae plants, especially *M*. *rubra*, is the primary source for extracting myricanol. The bark of *M*. *rubra* is a traditional remedy widely used in China and other Asian countries. Its medicinal use was first documented in the *Compendium of Materia Medica* (Bencao Gangmu) as “The decoction can be used for washing malignant ulcers and scabies, for gargling to relieve toothache, as well as for detoxifying arsenic poisoning and treating burns and scalds” [11]. This remedy is commonly used to treat tissue and bone injuries, burns, scalds, gastrointestinal ulcers and diarrhea [12]. Phytochemical analysis has shown that diarylheptanoids and flavonoids are the main active ingredients in the bark of *M*. *rubra*, with myricanol, myricanone, myricitrin, and myricitin identified as four of the most promising compounds. Research on the pharmacological activities and mechanisms of myricanol began around 2000, with most early studies focusing on in vitro antibacterial, antioxidant and anti-inflammatory cell assays [13]. Our group demonstrated that myricanol is the main active compound in the ethyl acetate fraction of *M*. *rubra* bark and contributes to the lipid-lowering activity of this fraction in obese C57BL/6J mice [10,14]. In 2019, a study found that myricanol ameliorated muscle dysfunction by activating deacetylase sirtuin 1 (SIRT1) and peroxisome proliferator-activated receptor γ coactivator 1α (PGC-1α), which generated widespread interest among researchers [15]. The same group further showed that myricanol stimulated lipid utilization and irisin production in the skeletal muscle of obese mice by modulating the AMPK/PGC-1α signaling pathway [16]. Our group confirmed that myricanol primarily affects glycerolipid metabolism, fat digestion and absorption, lipolysis, and lipid metabolism in mice [17]. The results of these animal studies indicate that myricanol may be a promising therapeutic agent for metabolic diseases.

**Figure 2:**
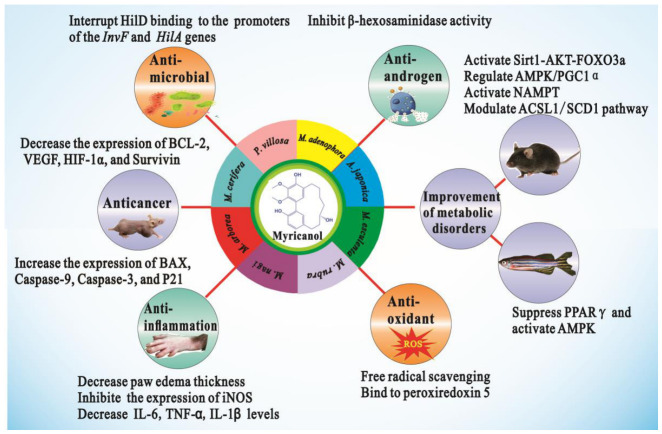
Primary plant source for extracting myricanol, its pharmacological activities, and underlying mechanisms. BAX: bcl-2-associated X protein; BCL-2: B-cell lymphoma 2; HIF-1α: hypoxia-inducible factor-1α; VEGF: vascular endothelial growth factor; AMPK: AMP-activated protein kinase; NAMPT: nicotinamide phosphoribosyltransferase; PGC-1α: peroxisome proliferator-activated receptor γ coactivator 1α; PPARγ: peroxisome proliferator-activated receptor γ; SIRT1: deacetylase sirtuin 1; AKT: protein kinase B; FOXO3a: Forkhead box O3a; ACSL1: long-chain acyl-CoA synthetase 1; SCD1: stearyl-coenzyme desaturase 1.

Since natural product-based drug discovery is crucial for new drug development, this review provides an overview of articles published in the past 26 years on the pharmacological effects and mechanisms of myricanol (Figure 2). Furthermore, the potential benefits, limitations, variability among published studies, and future research prospects of myricanol are discussed. This review will shed light on further investigations and applications of myricanol.

## 2. Materials and Methods

A comprehensive review of the published literature from scientific databases, including ScienceDirect, Web of Science, Google Scholar, and Chinese journal databases such as CNKI and Wan-Fang Data, was conducted using the keywords “myricanol”, “mechanism”, “separation”, “purification”, “activity” and “pharmacological activity” to gain insight into the bioactivities and mechanisms of myricanol. Articles published from 2000 to 2026 were reviewed to select studies that investigated the effects of myricanol in various experimental systems, including in vitro assays, cell culture models, and animal studies. Studies on myricanol derivatives, myricanol synthesis, and review articles were excluded from this review.

## 3. Pharmacological Effects and Mechanisms of Myricanol

Myricanol has been demonstrated to exert antioxidant, anti-inflammatory, anticancer, antimicrobial, and antihyperlipidemic activities, among others. Its role in metabolic modulation has been well studied in mouse and zebrafish models. However, most anticancer, anti-inflammatory and antioxidant research on myricanol has been conducted primarily in cell models. Several studies also indicate that myricanol has antifibrotic, antiallergic, and anti-atherosclerotic effects.

### 3.1. Antioxidant

The presence of two phenolic hydroxyl groups and one alcoholic hydroxyl group gives myricanol strong free radical scavenging properties. Recently, by comparing the 2,2-diphenyl-1-(2,4,6-trinitrophenyl)-hydrazyl (DPPH) free radical scavenging activity of myricanol, myricanone, porson and myricanol derivatives, Firmansyah et al. found that the free phenolic group at the C-5 position of myricanol significantly contributes to its reactive oxygen species (ROS) scavenging activity. The half-maximal inhibitory concentration (IC_50_) of myricanol for DPPH free radical scavenging is 39.3 µM, which is higher than that of myricanone and porson [18]. Myricanol at 0.42–0.84 mM significantly increased the viability of N2a cells against 100 µM H_2_O_2_-induced cytotoxicity. Additionally, myricanol treatment reduced H_2_O_2_-induced cytosolic Ca^2+^ overload in N2a cells [19]. A study published in 2010 reported that myricanol showed DPPH free radical scavenging with an IC_50_ of 14.9 µM [20]. Similarly, Gashaw et al. reported that the IC_50_ of myricanol for DPPH free radical scavenging is 13.48 µM [21]. Ting et al. showed that the concentrations required to scavenge 50% of the radicals (SC_50_) of myricanol for DPPH and ABTS were 198.9 µM and 22.3 µM, respectively [6]. Ibrahim et al. isolated myricanol from the stem bark of *A*. *japonica* and showed that the inhibition rate of myricanol at 50 μM for DPPH free radicals is 70.14% [7]. Intraperitoneal injection of myricanol at 10 mg/kg and 50 mg/kg for 20 days improved the muscle index, alleviated oxidative damage, and restored mitochondrial morphology and function in aged mice by down-regulating the expression of muscle-specific RING finger protein 1 (MURF1) and dynamin-related protein 1 (DRP1), and up-regulating the expression of myogenic differentiation 1, myogenin, uracil-DNA glycosylase 1 (UNG1), nuclear factor erythroid 2-related factor 2 (NRF2) and PGC-1α. Importantly, using large-scale pull-down and LC-MS methods, the authors demonstrated that myricanol can bind to the Cys100 residue of Peroxiredoxin 5 to relieve aging-related oxidative damage [3].

### 3.2. Anticancer

**Figure 3:**
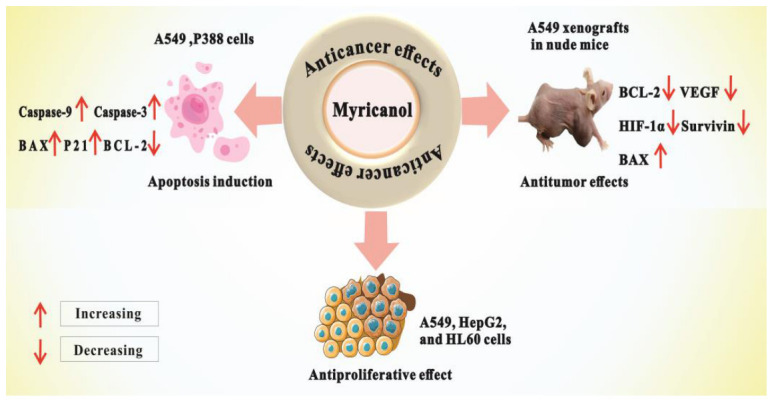
Anticancer effects and underlying mechanisms of myricanol in cancer cells, and nude mice. BAX: bcl-2-associated X protein; BCL-2: B-cell lymphoma 2; HIF-1α: hypoxia-inducible factor-1α; VEGF: vascular endothelial growth factor.

The anticancer activity of myricanol has been investigated in various cancer cell lines (Figure 3). Dai and colleagues demonstrated that myricanol inhibited the growth of A549 cells dose-dependently (IC_50_ = 13.53 μM) by upregulating the expression of Caspase-9, Caspase-3, BAX and P21, while downregulating BCL-2 expression [22]. This was the first report on the antitumor activity of myricanol, and the same group further examined its effects in nude mice. Administration of myricanol (10 mg/kg, 20 mg/kg and 40 mg/kg) to lung adenocarcinoma A549 xenografts in nude mice for 14 days significantly decelerated tumor growth, with inhibition rates ranging from 14.9% to 38.5%. Mechanistically, myricanol treatment decreased the expression of vascular endothelial growth factor (VEGF), BCL-2, Hypoxia-inducible factor (HIF), and Survivin, while increasing the expression of BAX, a pro-apoptotic protein [23]. Using isotope-labeling experiments, Dai et al. [24] showed that the stimulus index of 140 µM myricanol on A549, HepG2 and HL60 cells was 98.6%, 91.1%, and 95.9%, respectively. In another study, the IC_50_ values of myricanol against HL60, A549 and SK-BR-3 cells were 5.3 μM, 16.5 μM, and 14.8 μM, respectively. Myricanol increased the expression of Caspase-9, Caspase-3, and Caspase-8 in HL60 cells, indicating that it stimulates apoptosis through both the mitochondrial and death receptor pathways [25]. Additionally, the IC_50_ of myricanol against murine leukaemia P-388 cells was 21.5 μM [18]. A study on the structure–activity relationship showed that among 23 myricanol derivatives, 5-fluorobenzyloxy ether myricanol exhibited the strongest anticancer effect. Derivatives obtained by modifying both the 5 and 17 hydroxyl groups of myricanol were less effective in inhibiting A549 cells compared to those obtained by modifying only the 5-hydroxyl group. Moreover, modification of the 11-hydroxyl group significantly reduced the anti-cancer activity of myricanol [26].

### 3.3. Improvement of Metabolic Disorders

**Figure 4:**
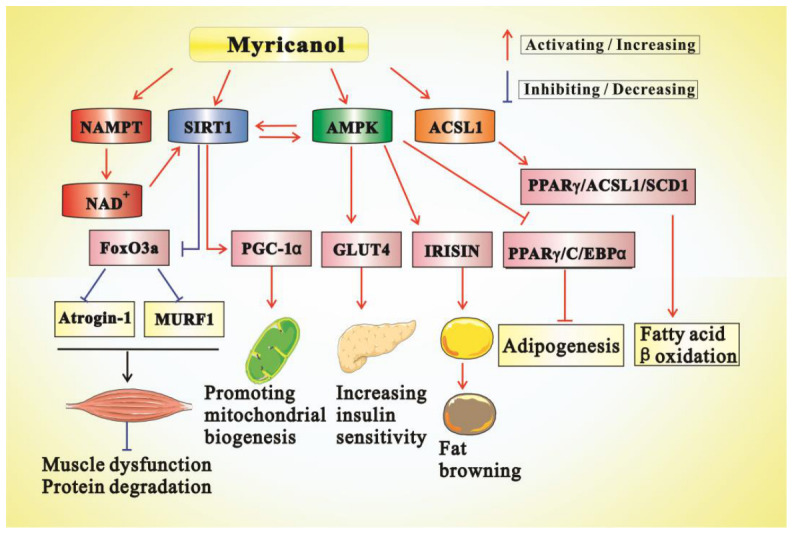
Effects and molecular mechanisms of myricanol in the management of metabolic disorders. AMPK: AMP-activated protein kinase; C/EBPα: CCAAT/ enhancer-binding protein α; GLUT4: glucose transporter type 4; MuRF1: muscle-specific RING finger protein 1; NAMPT: nicotinamide phosphoribosyltransferase; PGC-1α: peroxisome proliferator-activated receptor γ coactivator 1α; PPARγ: peroxisome proliferator-activated receptor γ; SIRT1: deacetylase sirtuin 1; ACSL1: long-chain acyl-CoA synthetase 1; SCD1: stearyl-coenzyme desaturase 1.

The beneficial effects of myricanol on metabolic disorders have been demonstrated in C57BL/6J mice and zebrafish models (Figure 4). Myricanol at 10 μM prevented dexamethasone (DEX)-induced muscle dysfunction and increased mitochondrial biogenesis in C2C12 myotubes. By binding to SIRT1, myricanol stimulated SIRT1 deacetylation, increased PGC-1αactivity, and decreased the transcriptional activity of forkhead box O3a. Intraperitoneal injection of myricanol (5 mg/kg, 50 mg/kg) in DEX-induced muscle atrophy in C57BL/6 mice for 10 days reduced muscle protein degradation, and enhanced autophagy and mitochondrial function by modulating various aspects of the SIRT1 signaling pathway [15]. In HFD-induced obesity mice, daily intraperitoneal injection of myricanol for 18 weeks promoted lipid utilization, induced browning of inguinal fat, and stimulated irisin production in skeletal muscle. These effects were attributed to regulation of the AMPK/PGC-1α signaling pathway by myricanol [16]. Shen et al. first discovered that myricanol serves as an activator targeting AMP-activated protein kinase (AMPK). Myricanol decreased lipid accumulation in 3T3-L1 cells by stimulating the insulin signaling pathway, inhibiting adipogenesis and promoting lipolysis and lipid oxidation. In HFD-fed zebrafish, treatment with 1.0 μM myricanol attenuated lipid accumulation by activating AMPK, while suppressing peroxisome proliferator-activated receptor γ (PPARγ) and CCAAT/enhancer-binding protein α (C/EBPα) [27]. Oral administration of myricanol at 100 mg/kg and 150 mg/kg for 30 days improved the metabolic profile of DEX-induced metabolic disorder in mice. Myricanol reversed muscle cell apoptosis and atrophy, increased muscle glucose utilization by increasing SIRT1 and glucose transporter type 4 (GLUT4) expression, and decreased MURF1 expression and Cleaved Caspase 3. Metabolomics revealed that myricanol treatment reversed the serum content of carnitine ph-C1, palmitoleic acid, four amino acids and a great number of glycerolipids in DEX-induced mice. Notably, metabolic pathway enrichment analysis revealed that myricanol mainly affects glycerolipid metabolism, lipolysis, fat digestion and absorption, and lipid metabolism pathways [17]. Nicotinamide phosphoribosyltransferase (NAMPT) is essential for the biosynthesis of nicotinamide adenine dinucleotide, a molecule critical to pancreatic β-cell function. Using pull-down assay and mass spectroscopy coupled with drug affinity responsive target stability assay, Lyu et al. revealed that myricanol enhances insulin-sensitizing activity and promotes glucose uptake in C2C12 myotubes by binding directly to NAMPT [28]. A recent study showed that myricanol administration reversed body weight and serum lipid levels in obese mice by modulating long-chain acyl-CoA synthetase 1 (ACSL1)-stearyl-coenzyme desaturase 1 (SCD1) axis and promoting mitochondrial biogenesis and fatty acid β-oxidation [29]. These in vivo studies indicate that myricanol holds great promise for the treatment of metabolic abnormalities.

### 3.4. Antibacterial Effect

*Salmonella typhimurium* (*S. typhimurium*) infection remains a threat to public health worldwide. Incubation of myricanol with *S. typhimurium* inhibited its invasion into SW480 cells in a dose-dependent manner by decreasing the expression of the pathogenesis-related *SPI-1* gene, with an IC_50_ of 41.37 µM. Further study revealed that myricanol can bind to HilD and interfere with HilD binding to the promoters of the *InvF* and *HilA* genes [30]. At 700 µM, myricanol showed moderate antibacterial activity against *S. aureus*, *S. pyogenes*, *E. coli*, and *P. aeruginosa*. Additionally, myricanol inhibited the growth of Gram-positive bacteria more efficiently than Gram-negative bacteria [21]. The minimal inhibitory concentration (MIC) value of (−)-myricanol against *Mycobacterium tuberculosis* H37Rv (*M. tuberculosis*) is 42.0 µM, which is higher than those of other pure compounds, including porson, 12-hydroxymyricanone, and myricanone isolated from the roots of *M. adenophora* [6].

### 3.5. Anti-Inflammation

The inorganic free radical NO is well known for its role in inflammation. In an LPS-induced mouse peritoneal macrophage inflammation model, myricanol showed an inhibitory effect on NO production with an IC_50_ of 23 µM. The inhibitory effect of myricanol on NO production was equivalent to that of NG-monomethyl-larginine (IC_50_ = 28 µM) and was attributed to suppression of inducible NO synthase (iNOS) expression [13]. In RAW 264.7 cells, myricanol decreased LPS-induced NO production with a median effective concentration (EC_50_) of 7.5 µM [6]. Similarly, myricanol was proven to be the main anti-inflammatory compound in the stem of *P. villosa*. In LPS-induced NO production in macrophage cells, 25, 50, and 100 μM myricanol decreased NO content by 16.0%, 55.6% and 75.5%, respectively, via the inhibition of iNOS expression. Pro-inflammatory cytokines, including IL-6, TNF-α and IL-1β, were also reduced by myricanol dose-dependently [5]. Of note, among five cyclic diarylheptanoids isolated from *A. japonica* stem bark, myricanol showed the highest anti-inflammatory activity in vivo. Administration of myricanol at 10 mg/kg significantly decreased paw edema thickness in a carrageenan-induced rat paw edema model, with inhibition rates of 31.6%, 44.3%, 51.5%, and 36.8% after treatment for 1, 2, 4, and 6 h, respectively [7].

### 3.6. Other Pharmacological Effects

In addition to the studies mentioned above, myricanol also exhibits anti-Alzheimer’s disease, anti-androgenic, antifibrotic, antiallergic and anti-atherosclerosis activities.

#### 3.6.1. Anti-Alzheimer’s Disease

Prevention of Tau protein aggregation is one of the most effective approaches for treating Alzheimer’s disease. Jones et al. found that the most powerful anti-Tau compound in bayberry was the diarylheptanoid myricanol, rather than myricitrin or myricetin. (+)-αR,11S-myricanol, isolated from *M. cerifera*, decreased Tau expression in HeLa-C3, IMR32 cells and mice brain slices dose-dependently, with an EC_50_ of 35 µM in HeLa-C3 cells [31].

#### 3.6.2. Anti-Androgenic Effect

Myricanol was reported to exhibit anti-androgenic activity. Among the four constituents of *Myricae cortex*, myricanol showed the highest testosterone 5α-reductase inhibitory activity, with an IC_50_ of 3.7 mM. In male Syrian strain golden hamsters, myricanol significantly decreased testosterone-stimulated growth of pigmented macules by 37%, similar to the effect observed in the oxendolone group [32].

#### 3.6.3. Antifibrotic Effect

Intraperitoneal administration of myricanol at 0.05 mg/kg and 0.2 mg/kg for one week alleviated renal fibrosis in a mouse model of chronic kidney disease by increasing mitochondrial transcription factor A (TFAM) transcription and subsequently promoting the interaction between zinc and ring finger protein 1 (ZNRF1) and lipocalin-2 (LCN2) [33].

#### 3.6.4. Anti-Allergic Effect

Matsuda et al. showed that both myricanol, (+)-S-myricanol, myricanone, myricanenes A and B, and myricetin from the bark of *M. rubra* exhibit considerable anti-allergic activity. Compared with the control group, (+)-S-myricanol at 10 µM, 30 µM, and 100 µM significantly reduced the activity of β-hexosaminidase in RBL-2H3 cells, with an IC_50_ of 28 µM [34].

#### 3.6.5. Anti-Atherosclerosis

Abnormal proliferation of vascular smooth muscle cells plays an essential part in the onset and progression of atherosclerosis and hypertension. Myricanol inhibited intimal hyperplasia after vascular injury in mice. Mechanistic studies showed that myricanol suppressed the phosphorylation of PDGFRβ and its downstream targets, including Phospholipase Cγ1 (PLCγ1), SRC, MAPKs and inhibited the nuclear translocation of NF-kB p65 [35].

## 4. Perspective and Discussion

Myricanol is emerging as a promising bioactive natural product. Its seven-carbon skeleton linked to two phenyl rings endows myricanol with multiple medicinal properties. This “privileged structure” can also be used in drug design and serve as a template for medicinal chemistry [36]. Several studies have demonstrated that myricanol is the main active compound in the bark of *M. rubra*, and its anti-inflammatory, protection against Alzheimer’s disease and testosterone 5α-reductase inhibitory activities are higher than those of myricanone, myricetin and other cyclic diarylheptanoids [7,31]. To obtain myricanol, the stem bark of the plant was first macerated in ethanol, methanol, or acetone. Because of its low polarity, myricanol can be further enriched by extraction with solvents such as chloroform, ethyl acetate, and hexane. The crude extract is then purified by silica gel column chromatography, medium-pressure liquid chromatography, or ODS column chromatography. Mixed solvents of hexane, ethyl acetate, and methanol were used as eluents to obtain myricanol [22,32]. Additionally, the chemical synthesis of myricanol has now been achieved. In 2010, Kawai et al. [37] demonstrated that both 4-coumaric acid and 3-(4-hydroxyphenyl) propionic acid were incorporated into the skeleton of myricanol. Martin et al. [38] established a synthetic route for myricanol using boronic acid pinacol ester and aryl iodide. Bochicchio et al. [39] accomplished the total synthesis of myricanol via a nine-step chemical reaction starting from methyl 3-(4-benzyloxyphenyl)propanoate.

According to the reviewed articles, the main pharmacological effects of myricanol are anticancer, anti-inflammatory, and antioxidant, as well as an improvement in metabolic disorders (Table 1). In particular, the reversal of metabolic abnormalities in obesity and type 2 diabetes mellitus by myricanol has been demonstrated in zebrafish and C57BL/6J mouse models [16,27]. However, most research on the anticancer, anti-inflammatory, and antioxidant properties of myricanol has been conducted using cell assays or chemical-based methods, which do not reflect the actual performance of the compound in vivo. Additionally, the variability observed in results from different studies, especially those concerning antioxidant and anticancer activities, may be due to diverse experimental conditions, including different chemical reaction conditions, cell lines, chiral configurations of myricanol, and solvents used for its dissolution. Therefore, standardized, well-established in vivo studies, such as lipid peroxidation assays for evaluating antioxidant activity [40] and tumor xenograft animal models for anticancer studies, should be used to investigate the bioactivities and mechanisms of myricanol.

**Figure 5:**
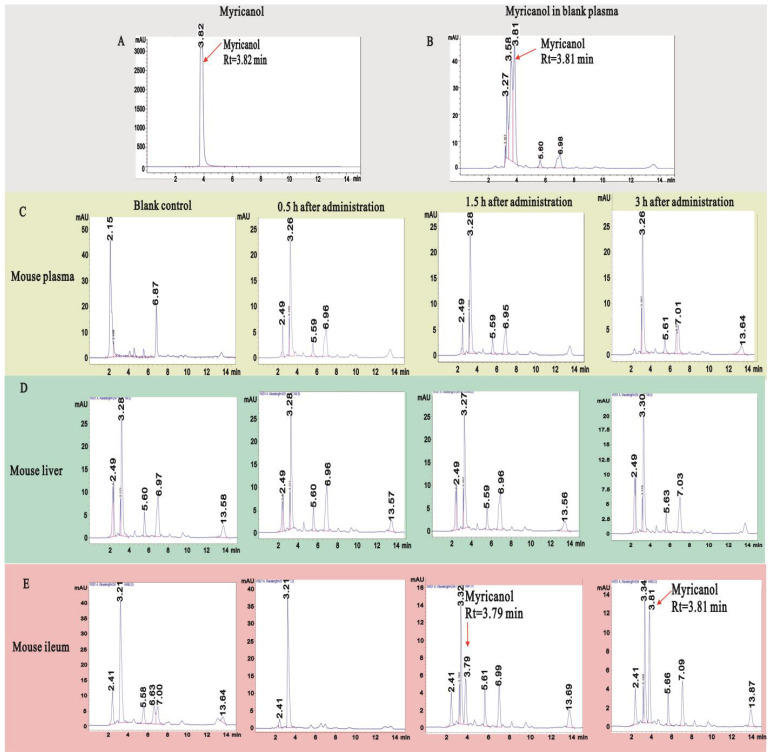
Myricanol detected using HPLC in mouse plasma, liver, and ileum at 0.5, 1.5, and 3 h after oral administration of myricanol at a dosage of 200 mg/kg. (**A**). HPLC graph of myricanol. (**B**). Myricanol in blank plasma. (**C**). Mouse plasma samples. (**D**). Mouse liver samples. (**E**). Mouse ileum samples.

Another challenge in developing myricanol for clinical use is a common limitation of diarylheptanoids: low oral absorption, bioavailability, and biodistribution [41]. To date, pharmacokinetic research on myricanol is lacking. Our study showed that no myricanol was detected by HPLC in the plasma or liver homogenates of C57BL/6J mice at 0.5, 1.5 and 3 h after oral gavage of myricanol at 200 mg/kg (limit of detection is 0.28 μg/mL). However, myricanol was detected in ileum samples collected at 1.5 and 3 h after administration (Figure 5). It has been reported that curcumin can be detected in mouse plasma at 15 min after oral administration, with plasma concentration reaching its maximum at 1.0 h and decreasing to the detection limit within 6 h [42]. This indicates that after oral administration, myricanol is mainly distributed in the mouse intestine, and its oral absorption and bioavailability are lower than those of curcumin. Whether myricanol is truly absent in mouse plasma and liver homogenates or present at levels below the limit of detection requires further investigation. The poor bioavailability of diarylheptanoids is attributed to their rapid metabolism in the liver and intestine. Studies have demonstrated that concomitant administration of piperine and curcumin increases the bioavailability of curcumin by 154% in rats and 2000% in healthy humans [43]. This is because piperine inhibits the activities of hepatic and intestinal UDP-glucuronosyltransferases and glucuronidation. Moreover, evidence shows that curcumin can affect the pharmacokinetics of many drugs by regulating intestinal CYP3A and P-gp [44,45]. To improve the bioavailability of curcumin, a simple and feasible approach is to combine curcumin with a CYP3A4 and P-glycoprotein blocker, such as piperine [46]. This method can also be applied to other diarylheptanoids, such as myricanol, as they share a similar core skeleton structure. There are two major ways to improve the bioavailability of myricanol. First, the hydroxyl, methoxy and carbonyl groups of diarylheptanoid compounds are suitable for specific chemical modifications, which can help researchers develop candidate drugs with improved solubility and bioavailability, and enhanced activity. For instance, three pyrimidinone analogs were obtained by replacing the two carbonyl groups of curcumin with a phenyl urea group. The product 1-(2,6-dichlorophenyl)-4,6-bis((E)-4-hydroxy-3-methoxystyryl) pyrimidin- 2(1H)-one showed stronger anticancer activity than curcumin, gefitinib, and imatinib against ovarian, prostate, breast, melanoma, and CNS cell lines [47]. The application of curcumin derivatives in cancer therapy has been reviewed [48]. Tetrahydrocurcumin and octahydrocurcumin, the hydrogenated metabolites of curcumin, exhibit more potent anti-hepatocellular carcinoma and hepatoprotective activities than their parent compound curcumin [48,49]. Another way to improve the druggability of myricanol is through pharmaceutical technologies. Sasaki et al. developed a submicron-dispersed formulation of curcumin called theracurmin, which is more bioavailable and effective than curcumin. A study showed that the bioavailability of theracurmin is more than 40-fold higher in rats and 27-fold higher in healthy human volunteers compared to curcumin [50]. Oral administration of theracurmin at 180 mg/day for 6 months prevented cognitive loss in Alzheimer’s patients [51]. Another well-known formulation of curcumin is BCM-95, which is composed of 95% curcuminoids and turmeric essential oil. The bioavailability of BCM-95 is about 7-fold higher than that of standard curcumin in humans [52]. Additionally, BCM-95 exerts a therapeutic effect in alleviating oral mucositis, dysphagia, and oral pain among patients with oral cancer [53].

It should be noted that myricanol is an enantiomeric compound (Figure 1), and its biological activity is closely related to its chiral structure. To date, most isolated myricanol samples have exhibited negative optical rotations, including myricanol isolated from the stem bark of *M*. *nagi* and the bacterial gall and bark of *M. rubra* [32,54]. However, Jones et al. isolated (+)-myricanol from the root-bark powder of *M. cerifera*. Intriguingly, (+)-myricanol significantly reduced Tau content in HeLa-C3 cells, while racemic myricanol showed no effect at the same concentrations [31]. Using enzymatic hydrolysis, (+)-S-myricanol was obtained from (+)-S-myricanol 5-O-β-D-glucopyranoside, which exhibited higher anti-allergic activity than myricanol (IC_50_: 28 µM vs. 63 µM) [34]. Therefore, further investigations are urgently required to compare the activity of individual myricanol enantiomers and to lay the foundation for its pharmacological study and clinical application. To date, several patents have been granted for the application of myricanol in the treatment of colitis, neurodegenerative diseases, and tumors [55,56,57]. Since myricanol is mainly distributed in the intestine, its pharmacological effects may be achieved by regulating intestinal flora and their secondary metabolites. Myricanol may have significant therapeutic potential for the management of gastrointestinal disorders such as peptic ulcers, diarrhea, and ulcerative colitis.

## 5. Conclusions

Myricanol exhibits a wide range of pharmacological activities, including antioxidant, anti-inflammatory, anticancer, antimicrobial, and antihyperlipidemic effects. It improves metabolic abnormalities in mice primarily by activating the AMPK/SIRT1/PGC-1α signaling pathway. Myricanol also regulates several key molecules, such as Caspase, the BCL-2 family, iNOS, ROS, and Peroxiredoxin 5, to exert its anticancer, anti-inflammatory, and antioxidant effects. Further research is needed to improve its bioavailability, confirm its pharmacological effects and mechanisms in vivo, and explore its pharmacokinetic properties and safety.

## Figures and Tables

**Table 1:** Summary of pharmacological effects and underlying mechanisms of action of myricanol.

Activity	Dose Range Tested	Study Model	Mechanism of Action	Reference
Anti-cancer	7.0/14/28 µM	A549 cells	Increasing Caspase-9, Caspase-3, BAX, and P21 expression; decreasing BCL-2 expression	[22]
	10/20/40 mg/kg	Lung adenocarcinoma A549 xenografts in nude mice	Decreasing BCL-2, VEGF, HIF-1α and Survivin expression; increasing BAX expression	[23]
	4.37–140 µM	A549, HepG2, and HL60 cells	Inhibiting cell proliferation	[24]
	IC_50_: 21.5 µM	Murine leukaemia P-388 cells	Inducing cell death	[18]
Antiinflammation	1–100 µM	Peritoneal macrophage; RAW 264.7 cells	Inhibiting iNOS expression; decreasing IL-6, TNF-α, IL-1β production	[5,6,13]
	10 mg/kg	Adult male albino rats	Decreasing paw edema thickness in rat paw edema model	[7]
Antibacterial	700 µM	*S.aureus*, *S.pyogenes*, *E.coli*, *P.aeruginosa*	Inhibiting bacterial growth	[21]
	MIC: 42 µM	Mycobacterium tuberculosis H37Rv	Inhibiting the growth of *M. tuberculosis*	[6]
	IC_50_: 41.34 µM	Salmonella Typhimurium	Inhibiting HilD from binding to the promoters of *InvF* and *HilA* genes	[30]
Antioxidant	840 µM	N2a cells	Inhibiting H_2_O_2_ induced intracellular ROS and Ca^2+^ overload	[19]
	IC_50_: 13.48–50 μM	DPPH Radical-Scavenging assay	Radical scavenging activity	[7,18,21,25]
	SC_50_: 198.9 μM for the DPPH radical; SC_50_: 29.3 μM for ABTS cation radical scavenging	DPPH Radical-Scavenging assay; ABTS cation radical scavenging	Radical scavenging activity	[6]
	10/50 mg/kg	C57BL/6J mice	Decreasing DRP1 expression; increasing UNG1, NRF2, and PGC-1α expression; binding to Peroxiredoxin 5	[3]
Improvement of metabolic abnormalities	1.25/2.5/5 μM for cell assay; 1 μM for animal study	3T3-L1 cells; zebrafish	Activating AMPK but suppressing PPARγ and C/EBPα	[27]
	100/150 mg/kg	C57BL/6J mice	Increasing SIRT1, GLUT4 expression; decreasing MURF1 and cleaved Caspase 3 expression	[17]
	2.5/5/10 µM for cell assay; 5/50 mg/kg for animal study	C2C12 myotubes; C57BL/6J mice	Activating SIRT1; increasing AKT and FOXO3a phosphorylation	[15]
	1.25/2.5/5 µM for cell assay; 0.05/0.25 mg/kg for animal study	C2C12 myotubes; C57BL/6J mice	Activating AMPK signaling pathway	[16]
	100/150 mg/kg	C57BL/6J mice	Modulating PPARγ/ACSL1/SCD1 metabolic signaling pathway	[29]
**Other effects**				
Anti-allergy	10–100 µM	RBL-2H3 cell	Inhibiting β-hexosaminidase activity	[34]
Anti-diabetes	5–40 µM	C2C12 myotubes	Binding to NAMPT	[28]
Anti-fibrosis	0.05/0.2 mg/kg	C57BL/6 male mice and CDH16-cre mice	Augmenting TFAM transcription; promoting ZNRF1 and LCN2 interaction	[33]
Anti-Alzheimer	50/100/250 µM	HeLa-C3 cells, IMR32 cells, murine brain slices	Decreasing Tau protein expression	[31]
Anti-atherosclerosis	3–30 μM for cell assay; 5 g/kg for animal study	Vascular smooth muscle cells; C57BL/6J mice	Suppressing PDGFRβ phosphorylation; inhibiting NF-kB p65 nuclear translocation	[35]

## Data Availability

All data are presented in the article.

## Abbreviations

The following abbreviations are used in this manuscript:

AKT	Protein kinase B
ACSL1	Long-chain acyl-CoA synthetase 1
AMPK	AMP-activated protein kinase
BAX	Bcl-2-associated X protein
BCL2	B-cell lymphoma 2
C/EBPα	CCAAT/enhancer-binding protein α
DEX	Dexamethasone
DPPH	2,2-diphenyl-1-(2,4,6-trinitrophenyl)-hydrazyl
DRP1	Dynamin-related protein 1
EC50	Median effective concentration
FOXO3a	Forkhead box O3a
GLUT4	Glucose transporter type 4
HIF-1α	Hypoxia-inducible factor-1α
HPLC	High-performance liquid chromatography
iNOS	Inducible NO synthase
LCN2	Lipocalin-2
MIC	Minimal inhibitory concentration
MuRF1	Muscle-specific RING finger protein 1
Nampt	Nicotinamide phosphoribosyltransferase
Nrf2	Nuclear factor erythroid 2-related factor 2
PGC-1α	Peroxisome proliferator-activated receptor γ coactivator 1α
PLCγ1	Phospholipase Cγ1
PPARγ	Peroxisome proliferator-activated receptor γ
ROS	Reactive oxygen species
SC50	Concentration required to scavenge 50% of the radicals
SCD1	Stearyl-coenzyme desaturase 1
SIRT1	Deacetylase sirtuin 1
TFAM	Mitochondrial transcription factor A
UNG1	Uracil-DNA glycosylase 1
VEGF	Vascular endothelial growth factor
ZNRF1	Zinc and ring finger protein 1
